# Determining the benefits and drawbacks of parents using personal connections and social networks for recruitment in research projects: a qualitative study

**DOI:** 10.1186/s40900-023-00470-1

**Published:** 2023-07-26

**Authors:** Laesa Kim, Carrie Costello, Michael A. Golding, Chloé Janse van Rensburg, Jennifer L. P. Protudjer, Kristy Wittmeier

**Affiliations:** 1 Family Liaison, BC Children’s Hospital Research Institute, 938 W 28th Ave, Vancouver, BC V5Z 4H4 Canada; 2 Children’s Hospital Research Institute of Manitoba, 715 McDermot Avenue, Winnipeg, MB R3E 3P4 Canada; 3 Department of Pediatrics and Child Health, Max Rady College of Medicine, Rady Faculty of Health Sciences, University of Manitoba, Winnipeg, MB Canada; 4 Department of Food and Human Nutritional Sciences, Faculty of Agricultural and Food Sciences, University of Manitoba, Winnipeg, MB Canada; 5 George and Fay Yee Centre for Healthcare Innovation, Winnipeg, MB Canada; 6 Institute of Environmental Medicine, Karolinska Institutet, Stockholm, Sweden

**Keywords:** Community networks, Patient-oriented research, Patient partner, Parent partnership, Parents, Recruitment, Qualitative research, Risks & benefits

## Abstract

**Background:**

It is becoming more common for parents of children with chronic conditions to join research teams as partners. Parent partnerships can help align research with what is relevant and important to families. It is also common for parent partners to be asked to share information about a study through their personal networks, which supports study recruitment. In this parent-led study, we explored parents' experiences when working together with researchers in patient-oriented research studies, in relation to study recruitment.

**Methods:**

Demographic data were collected through a brief online survey (SurveyMonkey®) and analysed descriptively (n, %, median (interquartile range; IQR)). Qualitative data were collected through focus groups and interviews (July to October 2021), transcribed verbatim, and analysed thematically. Parent co-leads were involved in every stage of the study, including study design, recruitment, data collection, analysis, interpretation, and knowledge mobilization.

**Results:**

Fifteen parents (n = 14 women) who had research partnership experience participated in this study. Most (n = 13) participants self-identified as White or of European descent. The majority (n = 10) had partnered in 1–3 research projects, while five participants had partnered in 4 + projects. Parents had a median of 3 years (IQR: 5) of partnership experience. We identified the following three themes: motivations, authentic partnerships, and learned decision making. Each theme included reflections about recruitment, and about research partnership in general. *Motivations* included a personal connection to the research topic, a connection to the community impacted by the research topic, and a desire to create change. *Authentic partnerships* were important for a meaningful experience, and enhanced participant’s willingness and ability to share study materials. *Learned decision making* reflected parents’ evolving decisions and practices related to sharing study information or personal information to support research. We provide a summary of participants’ recommendations for researchers who work with parent partners, and recommendations for parents as they approach research partnerships.

**Conclusions:**

Experiences shared by parents who have partnered in research provide valuable information to inform recruitment methods and improve team functioning. Parent partners expressed a willingness to support recruitment and valued a strong research team working together for a common outcome. This study yields a set of recommendations guiding future research that engages parents as team members.

**Supplementary Information:**

The online version contains supplementary material available at 10.1186/s40900-023-00470-1.

## Abstract (plain language summary)

It is becoming common for parents of children with chronic conditions to join research teams as partners. These partnerships help ensure that research is relevant to families. In partnership roles, parents are often asked to share information through their personal networks to support recruitment. In this parent-led study, we explored parents’ experiences related to sharing research information through their personal networks. Through interviews and focus groups, our team asked 15 parents with research partnership experience how they felt about using their personal connections to help recruit for research. Interviews and focus group recordings were transcribed. We looked at the data to find common patterns, or themes, and found three: motivations, authentic partnerships, and learned decision making. Each theme included reflections about recruitment, and about research partnership in general. *Motivations* included a personal connection to the research topic, a connection to the community impacted by the topic, and the desire to create change. *Authentic partnerships* were important for a meaningful experience, and enhanced participant’s willingness to share study recruitment materials. *Learned decision making* reflected parents’ evolving decisions and practices related to sharing study information or personal information to support research. We provide a summary of participants’ recommendations for researchers who work with parent partners, and for parents as they consider partnerships. Experiences shared by parents who have partnered in research provide valuable information to inform recruitment methods and improve team functioning. Parent partners expressed a willingness to support recruitment and valued a strong research team working together for a common outcome.

**Supplementary Information:**

The online version contains supplementary material available at 10.1186/s40900-023-00470-1.

## Background

Patient-Oriented Research is rooted in the idea that having patients inform research priorities and processes improves the relevance of the research and will ultimately enhance the ability of research to improve health outcomes [1]. In the child-health context, patient-oriented research projects often have parents^1^ and their child(ren) involved in research together, or solely involve parents for their perspective or as proxies for their child [2, 3]. Siblings, other family members, and children or adolescents without an accompanying family member are also engaged to bring their perspectives to research, though less often [2]. Each of these groups have unique and valuable perspectives to bring to research.

Parent partnership specifically, is a practice in research that has increased in recent years, helping to shape research priorities, methods, analysis, and knowledge translation [2–5], resulting in more meaningful projects. Parents are also commonly asked to provide support for recruitment as they can share study recruitment materials through their personal networks [2, 3]. This is an effective strategy, as enhanced recruitment via partner support is a consistently reported benefit of patient or parent engagement in research [2–4]. It is our team’s experience that some parents are specifically approached for research partnership due to their large personal or social networks. For online networks specifically, there is evidence that audience engagement is higher with research-related postings when these posts are shared through a pre-existing or established community, versus an online community that is developed for the sole purpose of engaging the public in research [6]. While there are clear benefits to ensuring that research recruitment efforts reach patient and parent networks, there is a gap in the research as to how this practice is experienced by the parent partners themselves. Multiple rubrics and frameworks exist to guide research partnership development and evaluation [7–12]. These can be further informed by an enhanced understanding of parent partners’ experience of research recruitment. The present study was developed as a collaboration between parent partner researchers and academic researchers to address this gap. We aimed to conduct a preliminary exploration of the benefits and drawbacks of using personal connections for recruiting research participants, as perceived by parents who have experience as research partners.

## Methods

Our team was led by two parent partners (CC, LK) with experience in patient-oriented research. The co-lead researchers are parents themselves to children with complex or chronic health conditions, much like our participants. Their lived experience aids them in their approach to research, including in the data collection, analysis, and interpretation. The research question was informed by their personal research partnership experience. The parent co-leads approached the academic researcher team members to collaborate on this work and provide methodological guidance. The team was further expanded to include a research coordinator and a student researcher. The team then co-created the study from methodology development to knowledge translation. Compensation for the co-leads was considered, and paid out of grant funds. The Guidance for Reporting Involvement of Patients and the Public 2 short form [13] was used to report parent partner involvement in our study (see Additional File 1).

### Participant recruitment

Participants were recruited in July 2021 via advertisements posted on the personal and institutional social media accounts associated with the research team members. Further recruitment occurred when some participants voluntarily shared study details with their personal networks following their interview or focus group. Prospective participants were required to have a child (of any age at the time of recruitment) with a chronic health condition, and have research partnership experience or have been approached to serve as a parent partner in research. The ability to participate in interviews in English was an inclusion criterion. When potential participants expressed interest, they received a study information letter and consent form. We aimed for a sample size of 15–20 participants for this work. Sample size was determined with pragmatic considerations in mind. Specifically, this sample size would allow for two to three focus groups of six to eight individuals each. This would provide comfortable group sizes for participation and the opportunity for a range of views to inform this early work in the area. This sample size range also was informed by the time and budget allocated for the project. This study was approved by the University of Manitoba Health Research Ethics Board (HS24672; H2021:077).

### Data collection

After providing consent, participants were asked to complete a brief demographics survey (Survey Monkey®) that included questions about their partnership experience. Race and ethnicity data were self-identified via open text responses in the survey. Qualitative data were collected through virtual focus groups and semi-structured interviews. These were held between July and October 2021 using Microsoft Teams®. Sessions were led by one of the two parent co-leads, who were both trained in qualitative interviewing by research team members with this expertise. One of the co-leads had prior experience and training in qualitative research through involvement in a different project. The interview guide was first developed by the two parent co-leads and revised through collaboration with the other team members, including two with formal training in qualitative research (see Additional File 2). The guide was primarily aimed at exploring the benefits and drawbacks of using personal networks to recruit for research, but it also contained questions regarding parent partners’ research experiences more generally, for contextual purposes. Acknowledging the potential for personal experience or bias to influence the discussion, the co-leads strove to remain empathically neutral and limited sharing their perspective within the discussion unless it was useful to generate or bridge discussion, clarify a question or comment, or prompt for elaboration of a participant’s response. Interviews were recorded and transcribed verbatim.

### Data analysis

Demographic data were analyzed descriptively (n, %, median, interquartile range (IQR); Microsoft Excel®). Qualitative transcripts were analyzed independently by three coders (LK, MG, CJvR) [14, 15]. Independent coders were used in this study to enhance the rigour of data analysis. Each coder brought unique knowledge and expertise to inform coding, which enriched the data analysis and interpretation. In addition to their common role of coding, each coder also had a unique role in the process. Michael (MG) is an experienced qualitative research coordinator who oversaw the process and advised on analysis methods. Laesa (LK) brought her lived experience perspective as well as prior qualitative research training and experience to inform the process and decision-making. She had also had familiarity with the data from leading a portion of the interviews and focus groups. Chloé (CJvR) had deep familiarity with the data as she attended all interviews and focus groups and transcribed the data in preparation for analysis.

Thematic analysis (Microsoft Word®) was used, which is a qualitative analysis technique used to identify and analyze patterns or themes in qualitative data. The approach to thematic analysis used in this study aligns with template analysis as described by Brooks and King [16–18]. Template analysis is a flexible approach to codebook thematic analysis that is well suited to use by teams, and for use with “real-world research” [16]. Our team used a hybrid inductive and deductive approach to analysis and theme generation [18, 19]. The analysts began by completing a preliminary coding of four transcripts. This involved applying short descriptions, or codes, to relevant sections or text. Preliminary codes were guided the by questions used in the interviews and focus groups, and the research questions. Additional codes were generated during analysis. Once complete, they met to discuss the results of initial coding, and developed a preliminary codebook which was subsequently shared with the full research team. After reviewing the codebook with the team, and incorporating feedback, the codebook was finalized and was used to code each of the transcripts. This included re-coding the initial four transcripts. When the coding process was complete, each analyst developed a series of draft themes based on an interpretation of their coding. The analysts then met to discuss this process and their preliminary themes, and then met with the team as a whole to finalize the themes and supporting quotes to be included in the manuscript.

## Results

### Participants

The study included 15 participants. Recruitment was closed after our minimum sample size was met, and no expressions of interest to participate were made for two consecutive weeks. Fourteen of the participants (93%) were women. Four participants took part in an individual interview, and nine participated in a focus group. Four focus groups were held in total to accommodate participants’ scheduling needs. Focus group size ranged from two to four participants. Participants had a median age of 46 years (IQR 12). Most (87%) participants self-identified as White or of European descent. Participants also self-identified as Chinese and Eurasian. Ten participants had partnered in one to three studies, and five participants had partnered in four or more. The stage of research where the most participants (67%) had experience was in developing the project idea. The stage that the least were involved in was data analysis (27%) (Table 1). Parents had a median of 3 years (IQR 5) of partnership experience.

**Table 1:** Participant involvement across select stages of research (n = 15)

Stage of research	Involvement in research stage (n, %)
Defining the project idea	10 (67%)
Informing the research methods	6 (40%)
Developing recruitment methods	7 (47%)
Actively recruiting participants	6 (40%)
Data analysis	4 (27%)
Knowledge translation	6 (40%)

Comparable numbers of participants reported their highest level of education to be a graduate degree (n = 6) or undergraduate degree (n = 5), with the remainder reporting professional degree, high school diploma, or college/technical diploma. Ten participants were currently involved in full-time or part-time work, and five were not currently involved in paid work or were on leave from paid employment. A majority (n = 8) reported an annual household income > $100,000, with the remaining participants reporting an income between $30,000 to $100,000. At the time of the study, participants lived in four different Canadian provinces. See Additional File 3 for additional family demographic information.

### Themes

Three overarching themes were identified: motivations, authentic partnership, and learned decision making. During discussions, it became apparent that participant perspectives about study recruitment were often intertwined with broader perspectives related to partnering in research. Accordingly, each theme includes participant views about parent partnership in research, as well as specific recruitment-related insights. See Table 2 for themes and additional representative quotes.

**Table 2:** Themes and representative quotes

Theme	Quotes
Motivations	“I really love finding out information. I love having a voice. I think I like the idea that research can move things forward and better life for families like mine.” (P3)
	“The value of the research being conducted is so powerful to me, I’m totally fine to put it out. I’m not tapping on shoulders; I’m generally putting it out to some Facebook groups I’m part of as part of the community.” (P15)
Authentic Partnership	“Where I didn’t feel like our partnership was authentic, I didn’t feel like my contributions were being respected, I didn’t feel good about sharing [recruitment material for] those because I didn’t feel great that participation in that study… was going to be… effective or something I could really endorse.” (P5)
	“The first barriers that I experienced were that nobody gave me a binder of what to do, what to expect, what is my role, what do words mean, what do all these acronyms mean, what is what, who is who and I really literally had to learn that accidentally and overcome my fear to ask really stupid questions.” (P6)
Learned Decision Making	“I would never post without permission, which is different than I did early on, and I have a lot of regrets about that, like I overshared, in kinda that… impulse to make change immediately.” (P5)
	“I don’t know that I’ll ever get to a point where I’m like ‘I’ve mastered it, everything I’m doing is perfect, I’ve got the right balance of sharing and not sharing and maintaining privacy and not maintaining privacy’ like we’ll never get there, it’s just constantly evolving and a constant learning process” (P9)

#### Motivations

##### Research partnership

Parents described being motivated to partner with a research team due to a personal connection to the focus of the research project, the positive feelings that accompany helping others, the existing connection or ability to connect to a community, and the opportunity to raise awareness and use their voice. “Let’s keep ensuring that the work is informed by the people it’s trying to help” (P15).

Parents talked about how their lived experience with their children draws them into research spaces. As one participant said, “This is personal to me” (P14). Sometimes parents were motivated by the possibilities for change that research might produce. And sometimes they were “driven by anger” (P3) and a desire to right the wrongs that they themselves have experienced or witnessed in the systems they navigate with their children.

##### Recruitment

Similar factors were described as motivating parents’ willingness to assist the research team with their recruitment efforts; if they were motivated to partner on a project, they were motivated to contribute and see that project through from beginning to end. “I was fine with it [sharing recruitment material] because if it wasn’t a project that I was interested in or passionate about I wouldn’t be involved in the first place” (P9). Parents indicated that compensation was not a driver in their motivations to partner, or recruit; however, a lack of compensation left them feeling undervalued, “I don’t want to be a part of anything that you're doing if you can’t respect my time” (P13) (see also: Authentic Partnerships). While over half of participants (n = 8) had experience assisting with recruitment (development of materials/active recruiting), some parents were unable to recruit online due to a lack of technological literacy or comfort on the various platforms. Parents reported that it was important for investigators to provide feedback on the effects of their partnership. Receiving this feedback allowed them to see the value in their contribution and furthered their motivation related to the project and partnership role.

#### Authentic partnerships

##### Research partnership

The authenticity of the research relationship was key to parents feeling invested in the project and valued as a team member. “I really think that establishing trust at first, is absolutely key, I mean absolutely fundamental to um the working, the quality of the working relationship.” (P6). Participants who experienced authentic partnerships reported that they felt genuinely valued, empowered to use their voice, were fairly compensated, had a clear conception of their role, and were encouraged to use their own unique skill set to contribute to the project. Participants indicated that authentic partnerships are better achieved when investigators are aware of, understand, and follow best practices for engagement.

In discussions of less authentic partnerships, participants described experiences of unclear expectations and power differentials. “There are so many barriers, like language and a discrepancy in power levels… and it is really hard to create trust in that atmosphere without equalizing the playing field and inviting people to the conversation as equals.” (P6). Inauthentic partnerships were also marked by inequality across the research team that was rooted in a lack of compensation, inadequate training, and too much institutional pressure and administrative hurdles; “Not honoring and respecting a person’s time in a way of compensation” (P13).

##### Recruitment

Parents indicated that when they were engaged in authentic partnerships, they were more willing to assist with all aspects of the project, including recruitment. Tangible indicators of an authentic research relationship related to recruitment included receiving guidance, tools, and feedback to assist with and refine recruitment efforts. In comparison, when participants and were not provided easily usable recruitment materials, nor feedback on their efforts, research relationships were viewed as less authentic.

#### Learned decision making

##### Research partnership

Within the final theme, Learned Decision Making, participants spoke about how parent partners learn from experiences, both within and outside of research, and used these learnings to inform partnership and recruitment-related decisions. The theme represents an evolution of practices over time. Experience led to changes in decision making when it came to choosing new research partnerships. Building on the previous two themes, some participants described learning about “who”, or which research team(s) they would or would not partner with in the future, and considered factors such as the authenticity of the relationship (if they partnered with a group in the past), and how closely the research aligned with their personal interests and goals to effect change.

##### Research recruitment

Parents who had been involved in more research studies, or had a longer duration of involvement described changes, or an evolution in how they used their social networks for recruitment, and a refinement of their approach to sharing information online. More experienced parent partners described being selective about where and how information is shared, considering who is the audience, and how often they go to these networks for recruitment purposes, “I think it is important to be very respectful of online groups and conversations and parents and say ‘is it permitted to discuss research here?’” (P6). Participants discussed the need to be aware of social media group etiquette, as there are often unwritten rules to consider when approaching a group, which can be dependent on whether the parent is already a part of this community or not. Some parents discussed their preference to share recruitment materials in relevant social media-based groups, rather than on their public or personal account. Reasons provided for this practice were to maintain their families’ privacy, and to ensure their efforts reach the intended audience.

Related to discussions about privacy, some of the participants with a longer duration of experience partnering in research expressed regret that they had overshared their families’ story when they were younger. The delineation of whose story was being told, or should be told, was not always clear. “it’s our story, it’s their story and it’s my story, and at what point, where are those lines?” (P8) With time, some parents refined their sharing to a more targeted audience or by limiting the amount of personal information they shared as a parent partner when assisting with recruitment effort. Some described a shift to talking only about their experiences as a parent, rather than talking about their child or family.

The discussion of social media permanence caused some to reflect that sharing recruitment materials as a parent partner can forever link a child or family to a given topic – whether it is relevant to their own diagnosis or not, given their connection to their parent. That being said, some parents who felt respected and engaged within their project team were comfortable with including personal messages when sharing the study recruitment materials.

**Fig. 1:**
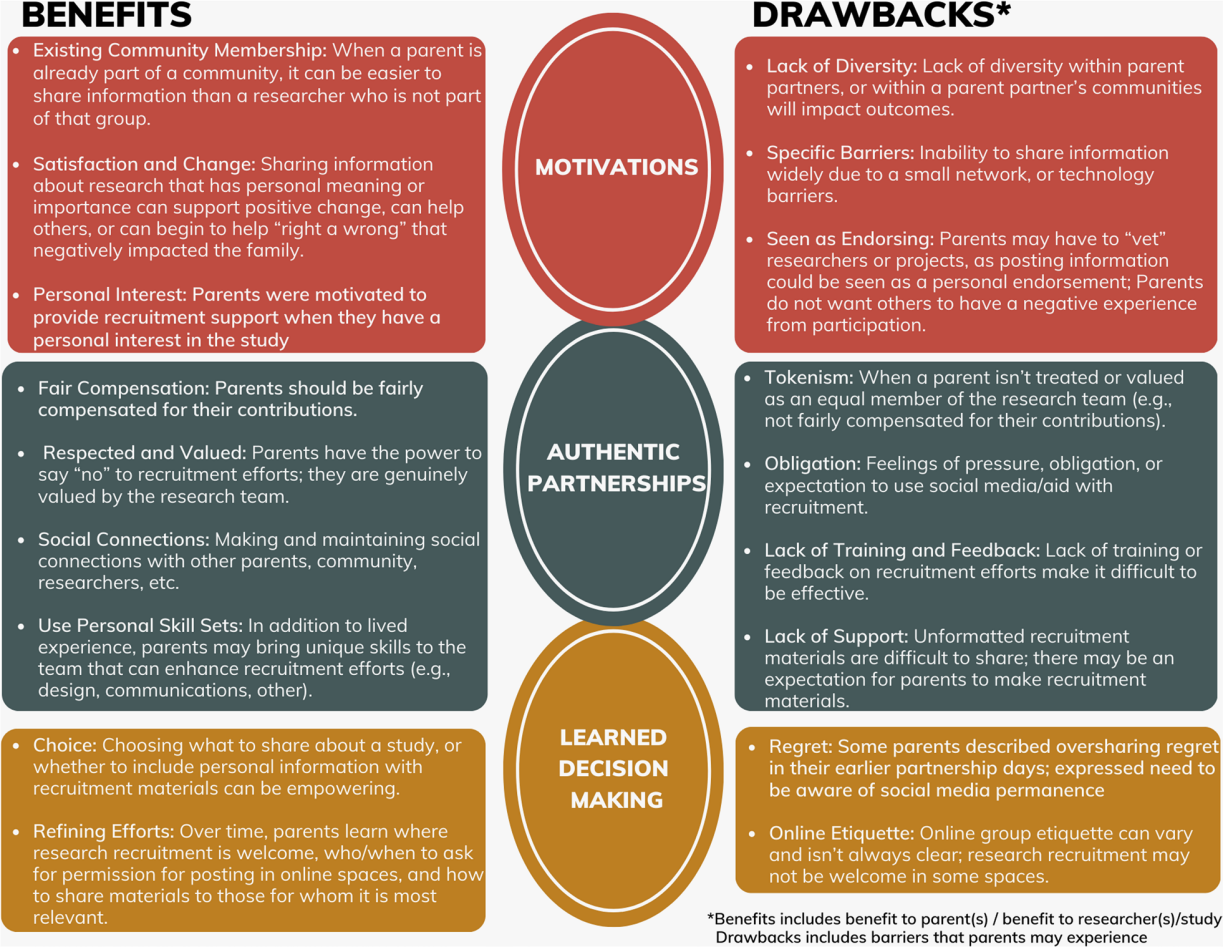
Benefits and drawbacks of parents using personal networks for recruitment organized by study themes

The relationship between the themes and the research question, specifically the benefits and drawbacks of using personal networks for research recruitment as discussed by participants, are presented in Fig. 1. Parents discussed benefits that related to the parent partner(s), to the researcher(s)/study, or both. The discussion about drawbacks included barriers that were experienced by parent partners.

## Discussion

Within this parent-led study, conversations about recruitment efforts could not be fully separated from broader conversations about research partnership. This underlines the need for each research partnership to be wholly authentic. The themes *Motivations, Authentic Partnerships,* and *Learned Decision Making* demonstrate the personal nature of research involvement and partnership, the drive and determination that parent partners hold as members of research teams, and the need for researcher team members to dedicate time, effort, and resources (e.g., compensation) to building trusting relationships. The decisions, skills, and practices of parent partners evolve over time, suggesting all team members should approach partnership as a dynamic practice that requires careful thought, planning, reflection, and sometimes a change of course. Finally, the relationship parents have with their child, their child’s condition or diagnosis, and the story they find themselves in, evolves over time, and for some, this evolution spurs them to approach both recruitment and partnerships more intentionally.

**Fig. 2:**
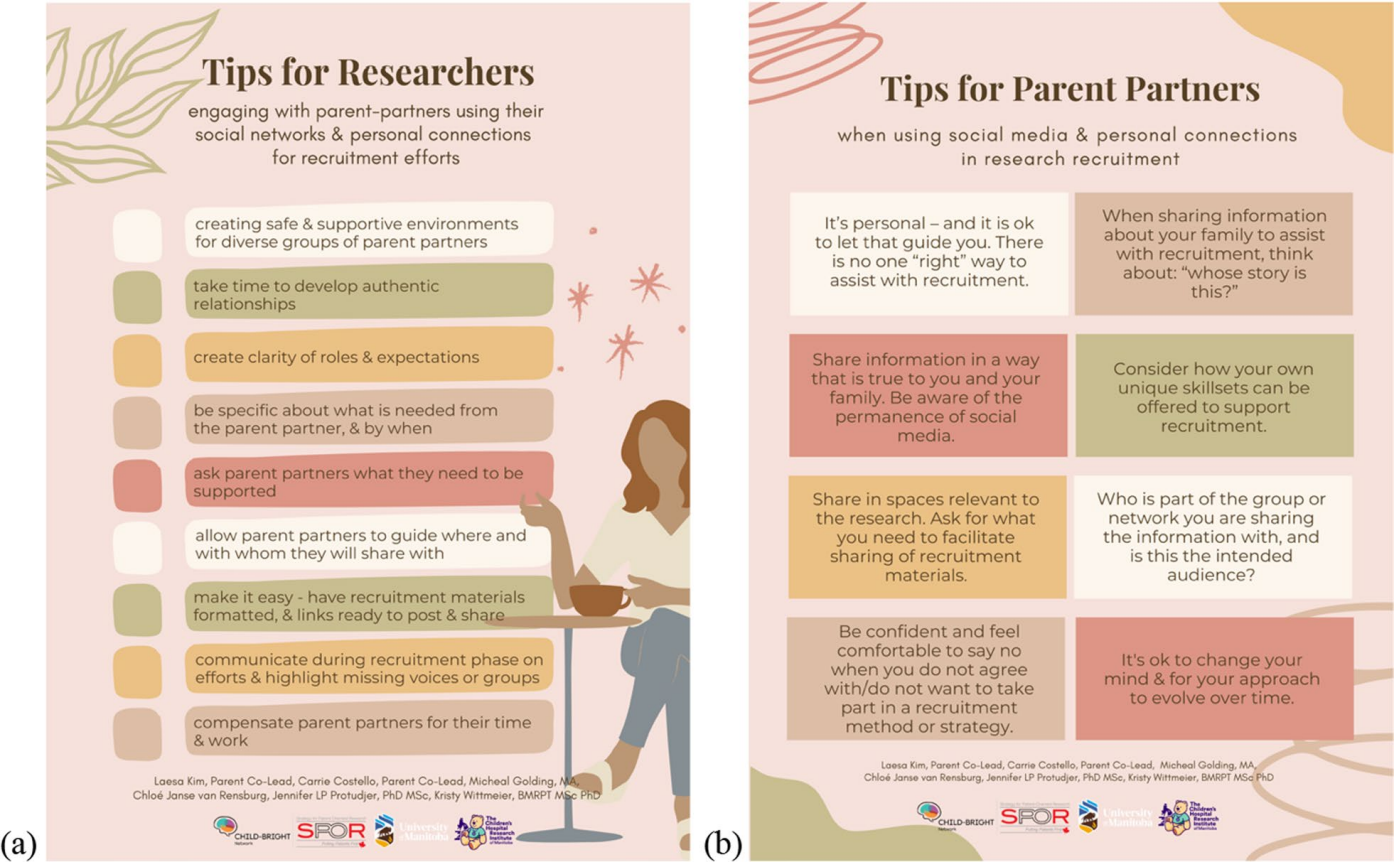
Tip sheets for researchers (**a**) and parent partners (**b**)

The overall evidence-base and guidance for patient/public engagement in research continues to grow [9, 20, 21], and includes enhanced guidance for partnership compensation [22, 23]. Similar to our findings, patient engagement frameworks and recommendations encourage research teams to dedicate time and resources to the development of strong, trusting, and respectful relationships [7, 9]. However, the experiences shared by participants, as well as those of the parent co-leads of this work suggest that there is still a need for knowledge, practical tools, and skill development to support the development of successful research partnerships, and to better support parent partners in recruitment efforts. Specifically, clear roles and expectations for all team members, appropriate compensation, open two-way conversations, as well as understanding and respect for the ethical and personal reasons parents may or may not want to share recruitment material were strategies that supported parents’ ability and willingness to recruit for research. The insights shared by participants are compiled into a list of recommendations for researchers and parent partners to consider, when working with each other to recruit participants through social media and other personal networks (Fig. 2; Additional File 4).

Our findings align with others who have reported on their research partnership experiences. Curran et al. shared “lessons learned” by their team over eight years of partnering with parents in research. These lessons included the need for transparency (e.g., regarding roles, expectations, timelines), respectful communication, and the creation of opportunities for meaningful engagement within a team [3]. Micsinszki and colleagues uniquely conceptualized a meaningful and authentic *invitation* to research partnership as “not just a tick box”, and a meaningful and authentic *participation experience* as “not just a rubber stamp” [24]. Both of these authors highlight the importance of understanding the unique motivations and skillsets of parents who are interested in partnering in research, and working to create clear roles suited to the skills and interests of partners. These factors were raised by participants in our study, associated with themes of “Motivations” and “Authentic Relationships”, in relation to research partnership, and in relation to supporting recruitment.

Similarly, Pozniak et al. reported on the experience of a parent-researcher partnership for the creation of a parent-focused workshop series [25]. They identified seven important components for a successful research partnership: consistent communication, clear roles and expectations, onboarding and feedback, flexibility, understanding, self-reflection, and funding [25]. These are in excellent alignment with our recommendations (see Tip Sheets, Fig. 2; Additional File 4) to support parent partners’ recruitment efforts. We contend that building strong research relationships will support the various roles that parent partners hold on a research team, including recruitment-related roles. For further guidance on practical suggestions to support the operationalization of Canada’s principles for patient-oriented research, we direct readers to the work of Santana and colleagues [26]. These authors also highlight the need for authentic relationship development and maintenance, clarity of roles and responsibilities, the need for communication and planning, and the importance of compensation. They present recommendations made by research partners to put the principles of inclusiveness, support, mutual respect, and co-building into practice [26].

As our study had a unique focus on parents' participation in recruitment, a novel aspect of our work was the exploration of decision making regarding what personal information to share in the context of research recruitment. Our findings highlight that even something seemingly simple like sharing a social media post can involve personal and ethical considerations for families. When a parent partner indicates their involvement in a study on social media, this may purposefully or inadvertently expose medical information about their child. Some families intentionally choose to share information online to raise awareness about a condition, or to help advocate for funding or for change. Others may prefer to limit these conversations within smaller circles. These can be nuanced decisions for families. We did not find in our sample that all parents retreated, or would advise retreating from sharing online, rather that their approach to sharing shifted. As described by the theme “Learned Decision Making”, changing how or what decisions were made over time represented a complex relationship between the parent and the story they found themselves in—their child’s or their own. The approach each family takes in sharing is deeply personal, and reflects their unique experiences, needs, and personhood at the time of sharing. Based on these findings, we encourage researchers and parent partners to have open discussions about the variety of ways parent partners can support recruitment, and to work together to create recruitment materials when possible. Parent partners should be supported to participate only in recruitment methods that suit their involvement goals and their comfort level. Researcher and clinician team members need to be aware of tacit feelings of obligation and be clear that sharing any recruitment material through any venue is optional.

Ironically, we found that our most effective way to recruit for the current study was to leverage the personal networks of the parent co-leads. This practice contributed to some limitations of the study, for example by limiting recruitment to known contacts and those in known networks. As this study took place while COVID-19 related restrictions were implemented in various provinces, recruitment occurred primarily online, through social media, and direct emails. Parents were required to have a device and sufficient internet/data for a video call, access to a phone/minutes on a mobile phone to participate. Most participants self-identified as White women, and over half of participants reported a household income of > $100,000. Further research with more diverse participant groups is essential.

This study also had a number of strengths. While the two methods of qualitative data collection introduced differences, it also facilitated data collection by allowing us to be flexible and accommodate participant preferences for timing and format. Parent participants who were involved in focus groups particularly expressed their appreciation for the opportunity to connect with other parents about their experiences in partnering in research. This suggests that opportunities for meaningful discussions between parents about parent partnership in general, including supporting each other in this type of work, may be lacking in the current research environment. Research networks that engage with parents, youth, and other partners with lived experiences can support these types of conversations by providing time, funding, and administrative support for research partners to debrief about experiences during and after involvement. Teams can also consider including team-based discussion as part of research partnership evaluations, to allow individuals to connect while sharing their perspectives about being involved in the research team.

Another strength of the study is that it is co-led by parents, who were involved in every stage of the research. The parent co-leads, who conducted the interviews and discussion groups, found that their self-identification as parent research partners helped participants feel comfortable in the conversation quickly. We also believe that this deepened the understanding of the interview and focus group dialogues, and the data that came from them. The pair complemented one another with differing views on the research questions at hand, and while there is always the possibility of bias, the co-leads mitigated this by remaining aligned with the interview guide created by the whole research team and having three team members coding, only one of whom is a parent partner. Both parent co-leads had been in proximity to some (less than half) of the participants, either personally or professionally, prior to the interview or focus group.

Of note, parent co-lead compensation for this project was considered and budgeted into a small grant that was received by the team. As the topic of compensation was present in this study’s findings, we will note here that the time dedicated to this work far exceeded the budget of the grant. This was in part balanced by using other research funds to support additional research coordinator time and student researcher participation. This topic will be explored further in a forthcoming evaluation of this research partnership.

As mentioned above, future research that involves a larger and more diverse participant group will be useful to build upon the findings reported herein. Use, feedback, and refinement of the recommendations put forward within this study for partners and researchers will contribute to efforts to build capacity in both groups, and support strong, effective, and authentic partnerships. The involvement of parents as partners in research can be daunting for both parents and researchers who have not previously engaged in partnered research, however it can be a rewarding and empowering experience for all, when done well [3, 27].

## Conclusion

This work examined the benefits and drawbacks of using personal connections for recruiting research participants, with parents who have served as research partners. The findings inform efforts to improve parent partnered research experiences, by identifying aspects of partnership and recruitment that are problematic for parents as well as those that are not. These considerations are important to ensure parent partners stay engaged with the research community and continue to offer their valuable contributions to the field.

## Supplementary Information

**Additional file 1.** GRIPP 2 short form.

**Additional file 2.** Interview guide.

**Additional file 3.** Additional family demographic information (n=15).

**Additional file 4.** Tip sheets for researchers and parent partners.

## Data Availability

Data are being retained at the primary research site (University of Manitoba); however, due to the small sample size and potential for identification, it will not be made available for sharing at this time.

## Abbreviation

IQR: Interquartile range

## Footnotes

1 Here, we use the term “parents” to collectively represent biological, adoptive and foster parents, as well as legal guardians.
