# Supplementary material for: Determining the benefits and drawbacks of parents using personal connections and social networks for recruitment in research projects: a qualitative study
Source: Res Involv Engagem. 2023 Jul 26;9:58. doi: 10.1186/s40900-023-00470-1 (PMC10373347; doi:10.1186/s40900-023-00470-1)
Supplement: Supplementary file 1 — Additional file 1. GRIPP 2 short form. [file 40900_2023_470_MOESM1_ESM.docx]

| **Section and topic** | **Item** | **Reported on page No** |
| --- | --- | --- |
| 1: Aim | Report the aim of PPI in the study | 7 |
| 2: Methods | Provide a clear description of the methods used for PPI in the study | 7,8 |
| 3: Study results | Outcomes—Report the results of PPI in the study, including both positive and negative outcomes | 19, 20 |
| 4: Discussion and conclusions | Outcomes—Comment on the extent to which PPI influenced the study overall. Describe positive and negative effects | 19, 20 |
| 5: Reflections /critical perspective | Comment critically on the study, reflecting on the things that went well and those that did not, so others can learn from this experience | 19-20 |

**Additional File 1: GRIPP 2 Short Form**
