# Supplementary material for: Determining the benefits and drawbacks of parents using personal connections and social networks for recruitment in research projects: a qualitative study
Source: Res Involv Engagem. 2023 Jul 26;9:58. doi: 10.1186/s40900-023-00470-1 (PMC10373347; doi:10.1186/s40900-023-00470-1)
Supplement: Supplementary file 2 — Additional file 2. Interview guide. [file 40900_2023_470_MOESM2_ESM.pdf]

## Additional File 2: Interview Guide

***Research team member:***

Thank you for meeting with me today. My name is (name) and I am one of the co-leaders on a project I'm conducting with researchers at the University of Manitoba to better understand how parents of children with chronic care needs perceive the benefits and drawbacks of using their own personal connections and networks to recruit other parents to research projects. Today, we are going to spend about 40-90 minutes talking about your involvement in patient-oriented research as a parent of a child with a chronic health condition. You may use names and places as you do in regular speech. However, we ask three things: First, that any information shared during this focus group is kept confidential and no identifying details are shared beyond this meeting. Second, be assured that, if you do refer to someone with details that could potentially identify them, we WILL NOT include such details in any reports or presentations. We will refer to you by a code and will never publicly say who you are. Third, this virtual meeting is considered a safe space, where everyone is encouraged to share as much or as little as they wish, and that no judgement will be made.

The process for this meeting is as follows: I will ask you a series of questions. Please answer them as best as you can. Some of the questions may make you a bit uncomfortable. Please consider answering them anyhow. But, if you prefer to not answer a question, or want to leave the discussion at any time, that is entirely your choice. You will not be penalized in any way. Do you have any questions before we start?

**Introductory questions**

1. To begin, I'd like to invite everyone to introduce themselves. Consider sharing your first name, what part of Canada you live in, some information about your family, and anything else you are comfortable sharing.

2. How did you hear about this study?

3. Have you ever used social media?

    a. If yes, what social media platforms do you use?

    b. In terms of your journey with your child's health, what do you use these various platforms for?

        Prompts: Do you connect with other families who have a child with a similar condition/diagnosis? Do you participate in advocacy groups? What information do you share on social media?

**Content questions**

1. What motivates you to partner in research?

2. What barriers to partnering in research have you encountered?

3. Have you been asked to use your personal social media accounts/networks to share information about a study or recruit for a study

   a. Whose idea was it to use your networks?

      Prompts: Did you offer to share information through your networks? Did the research team bring this up?

4. What were your initial thoughts about using your personal accounts/networks?

   Prompts: Did the research team ask you if you felt comfortable doing this? Did the research team mention it was okay to not participate in sharing the information?

5. If you have used your personal network for recruitment purposes, what aspects have worked well.

6. What has not worked well? (related to using social media/personal networks)

7. How do you feel now about using your personal accounts/networks for recruiting?

   a. Would you do this again? Why or why not?

   b. Would you do anything differently? Why of why not?

8. How do you decide as a parent how much you are willing to discuss about your family/child online? If your child is able to understand what you share online, how do you discuss this with them?

9. How has COVID influenced your motivation to participate in research?

10. How has COVID influenced your ability to participate in research?

**Wrap up**

11. What advice would you give to other parents who are considering using their personal social media networks to share recruitment information with others?

12. What advice would you give to researchers, who work with parent partners, regarding the practice of using personal social media networks for recruitment?

13. Is there anything else you would like to share about parents using their social media accounts/networks for research that we haven't covered?

**Summary**

- Now, I will provide a brief summary about what we talked about. Please correct me if anything is incorrect. (provide summary) How was that? Did I capture what you said or did I make some mistakes. (correct if necessary).
- Now that you've heard the summary, is there anything else that you would like to add?
- Thank you very much for your time and input to our study. We appreciate it.
