# Supplementary material for: Determining the benefits and drawbacks of parents using personal connections and social networks for recruitment in research projects: a qualitative study
Source: Res Involv Engagem. 2023 Jul 26;9:58. doi: 10.1186/s40900-023-00470-1 (PMC10373347; doi:10.1186/s40900-023-00470-1)
Supplement: Supplementary file 3 — Additional file 3. Additional family demographic information (n=15). [file 40900_2023_470_MOESM3_ESM.docx]

**Additional File 3: Additional family demographic information (n=15)**

| **Variables** | **Median (IQR)** |
| --- | --- |
| Number of adults living in the home | Median 2 (IQR 0.5) |
| Number of children living in the home | Median 2 (IQR 1) |
|  | **n (%)** |
| Number of children with one or more chronic health conditions*  1  >1 | 7 (43%)  8 (57%) |
| Child has received a diagnosis regarding their chronic health condition(s)*  Yes  No | 11 (79%)  4 (21%) |
| Relationship status  Married/common-law  Single/divorced/separated** | 12 (80%)  3 (20%) |

** Including, but not limited to autism, cerebral palsy, chronic pain, cystic fibrosis, intellectual disability, kidney disease, rare genetic conditions, and undiagnosed.*

***Categories combined due to low number*

**Abbreviation:** IQR: Interquartile range
