# Supplementary material for: Determining the benefits and drawbacks of parents using personal connections and social networks for recruitment in research projects: a qualitative study
Source: Res Involv Engagem. 2023 Jul 26;9:58. doi: 10.1186/s40900-023-00470-1 (PMC10373347; doi:10.1186/s40900-023-00470-1)
Supplement: Supplementary file 4 — Additional file 4. Tip sheets for researchers and parent partners. [file 40900_2023_470_MOESM4_ESM.pdf]

Tips for Researchers

engaging with parent-partners using their social networks & personal connections for recruitment efforts

- creating safe & supportive environments for diverse groups of parent partners
- take time to develop authentic relationships
- create clarity of roles & expectations
- be specific about what is needed from the parent partner, & by when
- ask parent partners what they need to be supported
- allow parent partners to guide where and with whom they will share with
- make it easy - have recruitment materials formatted, & links ready to post & share
- communicate during recruitment phase on efforts & highlight missing voices or groups
- compensate parent partners for their time & work

Laesa Kim, Parent Co-Lead, Carrie Costello, Parent Co-Lead, Micheal Golding, MA, Chloé Janse van Rensburg, Jennifer LP Protudjer, PhD MSc, Kristy Wittmeier, BMRPT MSc PhD

CHILD-BRIGHT Network
Strategy for Patient-Oriented Research SPOR Putting Patients First
University of Manitoba
The Children's Hospital Research Institute of Manitoba

Tips for Parent Partners

when using social media & personal connections in research recruitment

It's personal – and it is ok to let that guide you. There is no one "right" way to assist with recruitment.

When sharing information about your family to assist with recruitment, think about: "whose story is this?"

Share information in a way that is true to you and your family. Be aware of the permanence of social media.

Consider how your own unique skillsets can be offered to support recruitment.

Share in spaces relevant to the research. Ask for what you need to facilitate sharing of recruitment materials.

Who is part of the group or network you are sharing the information with, and is this the intended audience?

Be confident and feel comfortable to say no when you do not agree with/do not want to take part in a recruitment method or strategy.

It's ok to change your mind & for your approach to evolve over time.

Laesa Kim, Parent Co-Lead, Carrie Costello, Parent Co-Lead, Micheal Golding, MA,
Chloé Janse van Rensburg, Jennifer LP Protudjer, PhD MSc, Kristy Wittmeier, BMRPT MSc PhD

CHILD-BRIGHT Network
Strategy for Patient-Oriented Research SPOR Putting Patients First
University of Manitoba
The Children's Hospital Research Institute of Manitoba
